# Importance of hemodynamic RV and LV parameters and CPET-results in patients with Tetralogy of Fallot

**DOI:** 10.1186/1532-429X-17-S1-Q77

**Published:** 2015-02-03

**Authors:** Christian Meierhofer, Timon-Amir Tavakkoli, Andreas Kühn, Alfred Hager, Jan Müller, Stefan Martinoff, Peter Ewert, Heiko Stern, Sohrab Fratz

**Affiliations:** 1 Deutsches Herzzentrum München, Pediatric Cardiology and Congenital Heart Disease, Munich, Germany; 2 Deutsches Herzzentrum München, Division of Radiology, Munich, Germany

## Background

Good quality of life correlates with a good exercise capacity in daily life in patients with Tetralogy of Fallot (TOF). Patients after correction of TOF usually develop residual defects as pulmonary regurgitation or pulmonary stenosis of different severity. We investigated the impact of several hemodynamic parameters measured by cardiovascular magnetic resonance (CMR) and echocardiography and analysed these data together with results of cardiopulmonary exercise testing (CPET) of these patients.

## Methods

136 consecutive patients with TOF were tested during routine follow-up with CMR, echocardiography und CPET. Right and left ventricular volume data, ventricular ejection fraction, pulmonary regurgitation were evaluated by CMR. Echocardiographic pressure gradients in the right ventricular outflow tract (RVOT) and through the tricuspid valve area were measured.

**Figure 1:**
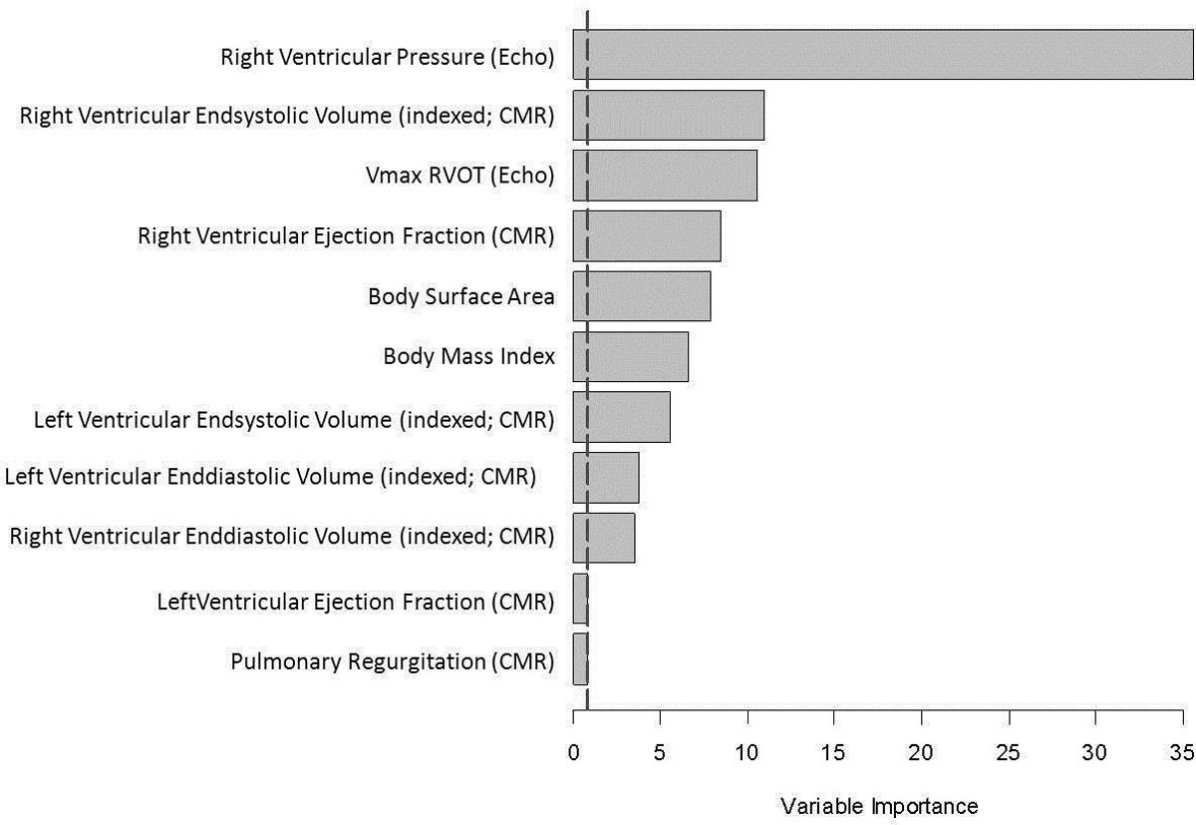


All data were classified and correlated with the results of CPET evaluations of these patients. The analysis was performed using the Random Forest model (classification and regression model with measurement of variable importance through permutation). In this way we calculated the importance of the different hemodynamic variables related to the maximal oxygen uptake in CPET (figure 1).

## Results

Right ventricular pressure showed the most important influence on maximal oxygen uptake, whereas pulmonary regurgitation and right ventricular end-diastolic volume were not important haemodynamic variables to predict maximal oxygen uptake in CPET.

## Conclusions

Patients with TOF and elevated right ventricular pressure showed a reduced exercise capacity. Maximal exercise capacity was only weakly influenced by right ventricular enddiastolic volume and not at all by pulmonary regurgitation.

## Funding

N/A.

